# Urine as a Noninvasive Diagnostic Approach for *Toxoplasma gondii*: A Systematic Review of Molecular and Serological Detection Methods

**DOI:** 10.1155/japr/9315889

**Published:** 2026-11-08

**Authors:** Bahareh Basirpour, Rohallah Abedian, Tooran Nayeri, Mitra Sadeghi, Shahabeddin Sarvi, Shaban Gohardehi, Seyed Abdollah Hosseini, Ahmad Daryani, Sargis Aghayan

**Affiliations:** ^1^ Toxoplasmosis Research Center Communicable Diseases Institute Mazandaran University of Medical Sciences Sari Iran mazums.ac.ir; ^2^ Department of Parasitology School of Medicine Mazandaran University of Medical Sciences Sari Iran mazums.ac.ir; ^3^ Student Research Committee Mazandaran University of Medical Sciences Sari Iran mazums.ac.ir; ^4^ Infectious and Tropical Diseases Research Center Dezful University of Medical Sciences Dezful Iran dums.ac.ir; ^5^ Laboratory of Molecular Parasitology Scientific Center of Zoology and Hydroecology Yerevan Armenia; ^6^ Laboratory of Zoology Research Institute of Biology Yerevan State University Yerevan Armenia ysu.am

**Keywords:** diagnosis, molecular, noninvasive, serodiagnosis, *Toxoplasma gondii*, toxoplasmosis, urine

## Abstract

**Background:**

Toxoplasmosis, caused by *Toxoplasma gondii* (*T*. *gondii*), is a globally prevalent parasitic infection affecting both humans and animals. Traditional diagnostic methods rely on invasive sampling techniques, including serum, cerebrospinal fluid, and tissue biopsies, which may pose challenges in clinical and research settings. This systematic review is aimed at evaluating the potential of urine as a noninvasive biological sample for the detection of *T*. *gondii* infection.

**Methods:**

A comprehensive literature search was conducted across five major international databases from September 1948 to October 2026. A total of 720 studies were initially identified, and after applying exclusion criteria, 21 eligible studies (10 experimental, 3 biochemical studies, and 8 descriptive) were included in this review.

**Results:**

All included studies successfully detected *T*. *gondii* DNA or antigens in both serum and urine samples using various serological (ELISA, RLA, and coagglutination) and molecular (PCR, nested‐PCR, qPCR, and LAMP) techniques. Notably, some studies reported superior detection sensitivity in urine compared with serum.

**Conclusion:**

This systematic review shows that urine is one of the promising, noninvasive alternatives for *T*. *gondii* detection and allows for easy and repeated sampling. Its noninvasive features, in comparison with invasive methods, encourage further clinical validation in an attempt to standardize diagnostic protocols based on urine.

## 1. Introduction

Toxoplasmosis is a zoonotic infection with a global distribution and a wide array of warm‐blooded hosts, including humans, which is caused by *Toxoplasma gondii*, an intracellular pathogenic parasite. Although felids serve as the definitive hosts of *T*. *gondii*, they can infect a broad variety of warm‐blooded animals, including humans. They can be transmitted when undercooked meat in which tissue cysts are present is ingested [1]. The seroprevalence of *T*. *gondii* in the world varies in a manner influenced by a multitude of climatic, public health, dietary, and economic factors. Approximately one‐third of the global human population has serum antibodies against *T*. *gondii*, indicating widespread exposure and a substantial reservoir of chronic infection [2]. Seroprevalence varies considerably between countries, with reported estimates ranging from 6.7% in South Korea to 68.6% in Brazil [3, 4]. Although infection in immunocompetent individuals is generally self‐limiting, it can cause severe complications in vulnerable populations. Primary maternal infection during pregnancy can result in congenital toxoplasmosis, potentially leading to miscarriage, fetal death, chorioretinitis, hydrocephalus, intracerebral calcifications, and neurological damage [3, 5, 6]. In immunocompromised individuals, particularly patients with acquired immunodeficiency syndrome (AIDS), reactivation of latent infection can cause life‐threatening toxoplasmic encephalitis [2, 3, 5].

The widespread burden of infection, together with the potentially severe consequences in vulnerable populations, underscores the importance of timely and reliable diagnosis to guide appropriate treatment. This clinical need also provides a rationale for diagnostic innovations that improve accessibility and reduce the burden of sample collection. Conventional diagnostic approaches commonly rely on blood, cerebrospinal fluid, or tissue samples, with collection procedures differing in invasiveness and associated risks. These considerations motivate the development of less invasive diagnostic approaches that can complement established methods while maintaining adequate diagnostic performance [6–8].

This systematic review will assess the existing literature on using urine samples for *T*. *gondii* detection by serological and molecular methodologies. A critical analysis of the strengths and weaknesses of these approaches using urine samples in terms of their feasibility and utility for toxoplasmosis diagnosis will allow this systematic review to identify their potential in being important patient‐centered alternatives to conventional methods.

## 2. Methods

### 2.1. Design and Protocol Registration

This review followed the Preferred Reporting Items for Systematic Reviews and Meta‐Analysis guidelines [9]. The protocol was registered on PROSPERO (CRD42020153541).

### 2.2. Search Strategy

A systematic literature search was performed using electronic databases, including ScienceDirect, Scopus, PubMed, and Web of Science from September 1948 to October 2025. Furthermore, Google Scholar was also searched to identify any additional studies that may have been missed the literature search was conducted to obtain studies on urine samples as noninvasive samples in the diagnosis of toxoplasmosis. Search terms are applied alone or in combination as follows: (“*Toxoplasma gondii*” OR toxoplasmosis) AND (urine OR urinary) AND (diagnos OR serodiagnos OR molecular).

### 2.3. Inclusion and Exclusion Criteria

#### 2.3.1. Inclusion Criteria

Inclusion criteria were as follows:

• Studies published in English
• Studies that investigated the detection of *T*. *gondii* in urine samples using various diagnostic methods, including serological, molecular, and culture‐based techniques.
• Both experimental and descriptive studies.

#### 2.3.2. Exclusion Criteria

Exclusion criteria were as follows:

• Studies that focused on the detection of *T*. *gondii* in biological samples other than urine (such as milk, sputum, blood, cerebrospinal fluid, and tissue samples).
• Letters, conference abstracts, gray literature, unrelated articles, review articles.
• Studies published in languages other than English.

### 2.4. Study Selection and Data Extraction

All of the studies were included in the EndNote software (Version X9, BLD 12062) and duplicate studies were removed. Two reviewers (R.A. and T.N.) evaluated the titles, abstracts, and full texts of the included studies. The third reviewer (S.A.H.) resolved possible controversies. The extracted data included the name of the first researcher, publication year of the study, place of study, number of samples, strain of *T*. *gondii*, diagnostic methods, type of gene marker, and results. Figure 1 represents a flowchart of the study design process.

**Figure 1:**
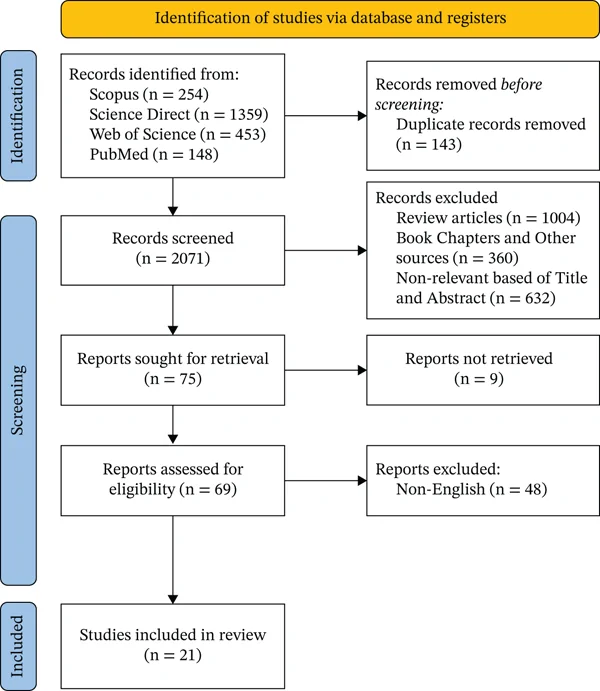
A flowchart describing the study design process.

## 3. Results

### 3.1. Study Characteristics and Search Results

The database searches identified 2214 records: 254 from Scopus, 1359 from ScienceDirect, 453 from Web of Science, and 148 from PubMed. After removing 143 duplicates, 2071 records underwent screening. Of these, 1996 were excluded, comprising 1004 review articles, 360 book chapters or other sources, and 632 records considered irrelevant based on their titles and abstracts. The remaining 75 reports were sought for full‐text retrieval. Six reports could not be retrieved, leaving 69 reports for full‐text eligibility assessment. Of these, 48 were excluded because they were not published in English. Ultimately, 21 studies evaluating urine‐based diagnosis of toxoplasmosis in laboratory animals (mice and rabbits; *n* = 11) and humans (*n* = 10) were included (Figure 1) from 1948 to October 2025. Of the 21 studies, 10 papers (47.61%) were experimental, 3 papers (14.28%) were biochemical analysis studies and 8 (38.09%) were cross‐sectional studies (descriptive). The detailed characteristics of the included studies are summarized in Tables 1, 2, and 3. Based on Tables 1, 2, and 3, the 10 experimental studies were conducted in six countries: Iran (*n* = 3), China (*n* = 2), the United States (*n* = 2), Belgium (*n* = 1), Cuba (*n* = 1), and Italy (*n* = 1); all three studies that analyzed the molecular and biochemical profiles of *T*. *gondii* infection in urine samples from animal models were conducted in China (*n* = 3), and the eight descriptive studies were conducted in seven countries: Spain (*n* = 2), Cuba (*n* = 1), Egypt (*n* = 1), Ghana (*n* = 1), India (*n* = 1), Saudi Arabia (*n* = 1), and the United States (*n* = 1).

**Table 1:** Baseline of experimental studies on urine samples included in this systematic review.

First author	Country	Host	Samples (*n*)	Positive (*n*)	Parasite strain	Methods	Gene markers	Serological results of serum	Serological results of urine	Molecular results of blood	Molecular results of urine	Molecular results of serum
Huskinson et al. 1989	United States	Mice (Swiss)	8	8	RH	ELISA	—	*T*. *gondii* antigens in sera of mice were demonstrated by ELISA at 4 days after infection	*T. gondii* antigens were demonstrable by ELISA in the urine of mice as early as 5 days postinfection.	—	—	—
Fachado et al. 1990	Cuba	Mice (BALB/c)	40	40	RH	Coagglutination	—	—	From the second day on all samples from infected mice were positive at 1/16 dilution.	—	—	—
Nguyen et al. 1996	Belgium	Mice NMRI	5	5	RH	PCR and DEIA	P30	—	—	Detection of *T*. *gondii* 3–4 days postinfection.	Detection of *T*. *gondii* 1 day postinfection.	
					Beverely	PCR and DEIA	P30	—	—	Detection of *T*. *gondii* 4–17 days in serum.	Not detected *T*. *gondii* in urine.	
Keshavarz and Shojaee, 2008	Iran	Mice (BALB/c)	50	20	RH	Capture ELISA		Detection of antigens of *T*. *gondii* in serum 3 days postinfection	Detection of antigens of *T*. *gondii* in urine 2 days postinfection.		—	
Hu et al. 2012	China	Mice	10	5	PRU	PCR and LAMP	B1	—	—		*T*. *gondii* was detected in urine samples from five surviving mice.	
Eteinberg et al. 2017	United States	Mice Swiss	8	4	RH	Hydrogel nanoparticle concentration, PRM, and Q‐PCR		—	—		Detected in 4 urine samples *T. gondii*, 4 days postinfection.	
				0	ME49	Hydrogel nanoparticle concentration, PRM, Q‐PCR		—	—		Not detected any *T*. *gondii* in urine samples.	
Luo et al. 2010	China	Mice	5	5	RH	Q‐PCR	B1	—	—		*T*. *gondii* was detected in all urine samples.	
Shojaee et al. 2008	Iran	Mice (BALB/c)	100	100	RH	PCR	B1	—	—		*T*. *gondii* was detected in urine 5 days postinfection.	
Eskandarian 2013	Iran	Human	10	10	RH	PCR	B1	—	—		*T*. *gondii* was detected in all urine samples.	
Cresti et al. 2001	Italy	HIV+ patients	2	2	RH	PCR‐nested and PCR	rDNA p30	—	—	*T*. *gondii* was detected in all two blood samples with rDNA gene.	*T*. *gondii* was detected in all two urine samples with rDNA gene and p30 gene.	

Abbreviations: DEIA, DNA enzyme immunoassay; ELISA, enzyme‐linked immunosorbent assay; LAMP, loop‐mediated isothermal amplification; PCR, polymerase chain reaction; PRM, parallel reaction monitoring; Q‐PCR, quantitative PCR.

**Table 2:** Baseline table of the studies that analyzed the molecular and biochemical profile of *T*. *gondii* infection in the urine samples of animal models.

First author (year)	Country	Host	Parasite strain	Diagnostic method	Result
Xie et al. (2022)	China	Japanese white rabbit	Pru	Sequencing noncoding RNA (miRNA and piRNA)	2089 miRNAs and 2224 novel piRNAs from the rabbit sera associated with *T*. *gondii* infection.
Zhou et al. (2022)	China	Mice (BALB/c)	Pru	Liquid chromatography‐tandem mass spectrometry (LC‐MS/MS)‐based metabolomics	Metabolites with altered levels in urine from *T*. *gondii*–infected mice revealed changes in pathways related to amino acid metabolism, fatty acid metabolism, and nicotinate and nicotinamide metabolism.
Cui et al. (2021)	China	Mice (BALB/c)	Pru	Liquid chromatography‐parallel reaction monitoring‐mass spectrometry (LC‐PRM‐MS)	A total of 13,414 peptides on 1802 proteins were identified, of which 169 and 47 proteins were signifcantly diferentially expressed at acute and chronic infection phases, respectively.

**Table 3:** Baseline of descriptive studies on urine samples included in this systematic review.

First author/year	Country	Studied population	Samples (*n*)	Positive (*n*)	Methods	Gene markers	Serological results of serum	Serological results of urine	Molecular results of blood	Molecular results of urine
Fachado et al. 1992	Cuba	HIV+ female	1	1	ELISA‐Western blot‐IFA‐rapid coagglutination	—	Detection of IgM, IgG anti–*T. gondii* antibodies with IFA method and detection of IgG with ELISA	Detection of antigens of *T. gondii* with rapid coagglutination method and Western blot	—	—
Fuentes et al. 1996	Spain	Infants	4	4	Nested PCR‐PCR‐hybridization, ELISA culture	B1	Detection of IgM (2 samples), IgG (4 samples), IgA (1 sample) anti–*T. gondii* antibodies with ELISA	—	Detection of *T. gondii* in 2 samples with PCR, in 3 samples with nested PCR, and hybridizations.	Detection of *T. gondii* in 3 samples with PCR, in 4 samples with nested PCR, and hybridizations.
Bhattacharyya et al. 2013	India	HIV+	100	42	ELISA	—	Detection of IgM (32 samples), IgG (42 samples) anti–*T. gondii* antibodies with ELISA	Detection of IgM (25 samples), IgG (42 samples) anti–*T. gondii* antibodies with ELISA	—	—
Mahmoud et al. 2002	Egypt	Human	40	39	Capture ELISA‐RLA	—	Detection of antigens of *T. gondii* in 38 samples with RLA and in 29 samples with capture ELISA methods	Detection of antigens of *T. gondii* in 39 samples with RLA and in 30 samples with capture ELISA methods	—	—
Ayi et al. 2004	Ghana	Ocular	54	47	DAT‐LAT			Detection of anti–*T. gondii* antibodies in 47 samples with DAT and in 46 samples with LAT		
		Pregnant women	26	19	DAT‐LAT			Detection of anti–*T. gondii* antibodies in 19 samples with DAT and in 17 samples with LAT		
Fuentes et al. 2001	Spain	Pregnant women	1	1	Nested‐PCR and PCR	B1				Detection of *T. gondii* DNA in urine samples with nested PCR
Elsamoual et al. 2020	Saudi Arabia	Women	22	18	LAMP	B1			8	10
Steinberg et al. 2021	United States	HIV positive patients	164	121	QPCR, PRM, and Western blotting technique		121		10 qPCR positive	30 qPCR positive and 8 PRM positive

Abbreviations: DAT, direct agglutination test; ELISA, enzyme‐linked immunosorbent assay; IFA, indirect immunofluorescence assay; LAMP, loop‐mediated isothermal amplification; LAT, latex agglutination test; PCR, polymerase chain reaction; PRM, parallel‐reaction‐monitoring; RLA: revers latex assay.

### 3.2. Findings of Experimental Studies on Human Urine Samples and Animal Models

A total of 140 out of 238 urine samples were analyzed using various molecular techniques, including polymerase chain reaction (PCR) [6, 8, 10–12], nested PCR [12], quantitative polymerase chain reaction (qPCR) [13, 14], loop‐mediated isothermal amplification (LAMP) [6], and DNA enzyme immunoassay (DEIA) [15] to detect *T*. *gondii* DNA. Moreover, B1 gene was the most frequently targeted marker in multiple studies [6, 8, 11, 14, 15], whereas rDNA and P30 were used in another specific studies [12, 15].

Additionally, three studies (98 out of 238 samples) exclusively employed serological methods, such as enzyme‐linked immunosorbent assay (ELISA), coagglutination, and capture ELISA [10, 16, 17] (Table 1). Furthermore, there are many studies illustrating comparable diagnostic performance between serum and urine, with some reporting faster detection in urine including Cresti et al. [12] and Keshavarz and Shojaee [10]).

There are several experimental studies focused on analyzing the molecular and biochemical profiles of *T*. *gondii* infection in urine samples from laboratory animals. As shown in Table 2, one study identified the noncoding RNA (including miRNA and piRNA) profile of *T*. *gondii* infection in the urine of Japanese white rabbits [18]. Another study investigated urinary proteomics during *T*. *gondii* infection [19], whereas a separate study explored the metabolomic profile of *T. gondii* infection in the urine of BALB/c mice [20].

Among the molecular techniques employed for *T*. *gondii* DNA detection, PCR, nested PCR, and qPCR demonstrated superior sensitivity, successfully identifying *T*. *gondii* DNA in all infected mice [11, 12, 14]. Regarding serological methods, ELISA and coagglutination exhibited the highest diagnostic accuracy, detecting *T*. *gondii* antigens in all tested samples [15–17].

Various *T*. *gondii* strains were used in experimental studies. The RH strain was the most frequently utilized, appearing in seven studies [8, 10–12, 14, 16, 17]. The PRU strain was used in four studies [15, 18–20]. Additionally, the RH and Beverley strains were used in combination in one study [15], whereas another study employed both the RH and ME49 strains [13] (Table 1).

### 3.3. Findings of Descriptive Studies Based on Human Urine Samples

In cross‐sectional studies, four studies investigated the status of *T*. *gondii* in both urine and serum as well as two studies carried out only on urine (Table 3). In addition, five of these studies were conducted on healthy individuals (such as pregnant women, infants, and the general population) and three of these studies included HIV‐positive persons. A total number of 412 samples were examined using different molecular and serological tests. The most studies were performed in Spain [21, 22], followed by Cuba [23], Egypt [24], Ghana [25], and India [26]. Four studies used serological methods including ELISA, Western blot, IFA (indirect immunofluorescence assay), rapid coagglutination, RLA (reverse latex assay), DAT (direct agglutination test), and LAT (latex agglutination test) [23–26]. One study used only molecular methods, such as PCR and nested PCR [22]. One study used only molecular methods, such as LAMP [27]. One study used only molecular methods, such as hydrogel nanoparticles, PRM, and QPCR. In addition, nested PCR, hybridization, ELISA, and Culture were used in one study [21]. The gene used in these studies was B1 [21, 22].

## 4. Discussion

Toxoplasmosis is a zoonotic infection that causes significant clinical manifestations, particularly in immunocompromised individuals and pregnant women. Despite its global prevalence, diagnosing toxoplasmosis remains a considerable challenge [28]. Conventional diagnostic approaches, including serology, PCR, histology, and culture, commonly require blood, cerebrospinal fluid, or tissue samples. Collecting these specimens can cause discomfort and, particularly for cerebrospinal fluid and tissue sampling, introduce procedural risks [29]. Urine offers a noninvasive alternative that may reduce the burden of sample collection and improve the accessibility of diagnostic testing [8, 29].

This systematic review examined experimental and descriptive studies evaluating urine‐based detection of *T*. *gondii* antigens and DNA. Although the findings support further investigation of urine as a diagnostic specimen, differences in detection timing, assay methods, parasite strains, and clinical populations limit conclusions about its reliability as a replacement for established tests. The findings are therefore discussed in relation to early detection, diagnostic methods, strain variability, and clinical applicability.

### 4.1. Early Detection Potential

Experimental studies suggest that urinary detection may be possible early in infection, although its timing relative to serum detection is inconsistent. Keshavarz and Shojaee [10] and Nguyen et al. [15] reported urinary detection at 2 days and 1 day postinfection, respectively. In contrast, Huskinson et al. [16] reported urinary antigen detection around 5 days postinfection, later than detection in serum. These contrasting observations suggest that urine does not consistently provide an earlier diagnostic signal than serum.

The potential value of early urinary detection lies in obtaining diagnostic information without invasive sampling. However, the earliest positive result in an experimental model does not establish a reliable diagnostic window in humans. Differences in infection stage, assay sensitivity, parasite strain, and host model must be considered when interpreting these findings. Urine and blood should therefore be compared at matched time points before any advantage in early detection can be established.

### 4.2. Antigen Detection and Molecular Assays

Urine‐based methods offer different practical advantages depending on whether they detect parasite antigens or DNA. Rapid antigen‐detection assays may be useful where short turnaround times and simple procedures are priorities. Fachado et al. [17] detected urinary *T*. *gondii* antigens in a patient with AIDS using rapid coagglutination, with a high speed. Similarly, Mahmoud [24] reported results within 2–3 minutes using RLA, with reported sensitivities of 97.5% for RLA and 75% for ELISA in urine samples. These findings suggest practical advantages for rapid testing, but speed alone does not establish diagnostic accuracy or suitability for routine use. Moreover, Ayi et al. [25] reported higher diagnostic performance for DAT (82.5%) than LAT (78%) in urine samples from immunocompromised individuals. However, comparisons between assays require clarity about whether the reported percentages represent sensitivity, positivity rates, or another performance measure. These measures should not be treated as interchangeable when interpreting the diagnostic value of urine.

Molecular approaches, including PCR, nested PCR, qPCR, and LAMP, provide an alternative means of detecting *T*. *gondii* DNA. Shojaee et al. [11] and Hu et al. [6] reported urinary DNA detection at 5 and 90 days postinfection, respectively, illustrating the range of sampling times represented in the experimental evidence. The B1 gene was a commonly used molecular target in the reviewed studies. However, its frequent use does not establish that it is the most sensitive target without direct comparisons under equivalent testing conditions.

In a human study, Ahmedani et al. [27] reported LAMP positivity in 90.90% of urine samples and 72.72% of blood samples from women. Although this finding supports further evaluation of urine‐based molecular testing, a higher positivity rate does not by itself demonstrate greater diagnostic sensitivity; that conclusion requires comparison with an appropriate reference standard. Overall, molecular and antigen‐detection assays should be evaluated separately rather than interpreted as a single category of urine‐based testing.

Serologic cross reactivity of *T*. *gondii* with *Hammondia hammondi*, *Neospora caninum*, and other related parasites has been documented in various studies [30]. Also, in the presence of an autoimmune inflammatory condition, which produces rheumatoid factor, false‐positive anti–*T*. *gondii* IgM results may occur due to nonspecific assay interference. Since IgM ELISA and antigen ELISA are different assays measuring distinct analytes, and cross reactivity and interference may not be the same, results of these assays need to be interpreted appropriately [31]. Thus, positive urine‐based ELISA results should be interpreted with caution, especially when the primary studies do not adequately assess the specificity of the assay, or positivity is not validated independently. Although the true impact of these mechanisms in the primary studies included in this review is speculative, inadequate assessment of assay specificity creates a concern regarding the diagnostic performance of the interventions being evaluated.

### 4.3. Strain Variability and Experimental Context

Parasite strain may contribute to differences in urinary detection. Steinberg et al. [13] detected urinary antigens at 4 days postinfection with the RH strain but did not detect antigens with the ME49 strain under the conditions examined. This contrast suggests that findings obtained with one strain should not automatically be generalized to others.

Differences in parasite replication, virulence, and cyst formation could help explain variation between RH, ME49, PRU, and Beverley strains, but these explanations remain hypotheses unless directly tested. One of the limitations of the current reviewed observations is not establishing a consistent ranking of strains by detection timing because strain differences may be confounded by assay choice, sampling schedule, and experimental design. Likewise, detection across BALB/c, Swiss, and NMRI mice does not demonstrate that host background has no effect. These sources of variability should be considered when translating experimental findings into clinical diagnostic strategies.

### 4.4. Clinical Applicability in Immunocompromised Patients

The descriptive studies suggest that urine‐based testing warrants particular attention in immunocompromised patients, including those with suspected toxoplasmic encephalitis. Its principal potential advantage is the availability of a noninvasive specimen that could complement existing diagnostic investigations. However, promising findings from individual patients or selected clinical groups do not establish performance across broader populations.

Steinberg et al. [32] evaluated 164 hospitalized HIV‐positive patients with neurological symptoms compatible with toxoplasmic encephalitis. Urinary GRA1 and SAG1 antigen testing yielded positive results in 30/164 and 12/164 patients, respectively. In a subgroup of 44 patients, parallel reaction monitoring mass spectrometry identified antigens in eight urine samples, six of which were concordant with the urine diagnostic assay [30]. These findings support the feasibility of urinary antigen detection in this clinical setting, but the different numbers of patients undergoing serology, blood qPCR, and cerebrospinal fluid qPCR prevent a simple comparison of positivity rates across methods.

Taken together, the human studies support urine as a promising complementary specimen rather than an established substitute for serum, blood, or cerebrospinal fluid. Clinical validation should distinguish infection detection from diagnosis of active disease and assess performance in clearly defined patient groups using an appropriate reference standard. The current evidence does not justify assuming equivalent performance in immunocompromised patients, pregnant women, and other populations.

Overall, urine‐based testing offers a potentially accessible and less burdensome approach to toxoplasmosis diagnosis. Its clinical role will depend on establishing reproducible performance across assay platforms, infection stages, parasite strains, and patient populations. Until these issues are resolved, the strongest interpretation is that urine may complement established diagnostic methods rather than replace them.

### 4.5. Limitations

#### 4.5.1. Study Design Limitations

The included studies varied in sample size, host species, and methodology. Small sample sizes in some studies and differences in study design limit the comparability of the findings and their generalizability. Restricting eligibility to English‐language publications may also have introduced language bias by excluding relevant local evidence, including studies from Latin America, China, and Eastern Europe. Consequently, the included studies may not fully represent the geographical diversity of the available evidence. The extent and direction of any resulting bias remain uncertain because non‐English studies were not assessed.

#### 4.5.2. Diagnostic Limitations

The absence of standardized diagnostic protocols and differences in *T*. *gondii* strains complicate comparisons across studies. Molecular techniques, particularly PCR, may yield false‐negative results, whereas contamination during sample collection or processing may produce false‐positive results. These concerns highlight the importance of standardized sampling procedures, assay optimization, and appropriate quality controls. Furthermore, limited comparisons with established diagnostic methods prevent firm conclusions about the relative performance of urine‐based tests.

#### 4.5.3. Evidence Gaps

The lack of longitudinal studies limits understanding of how urinary antigen and DNA detection change over the course of infection. Insufficient clinical validation also restricts conclusions about the usefulness of urine‐based testing in routine practice. Larger, well‐designed studies using standardized protocols and appropriate reference standards are needed to establish diagnostic performance across populations and clinical settings. Future reviews should also incorporate multilingual searches and, where feasible, translation of potentially eligible studies to improve evidence coverage.

## 5. Conclusion

This systematic review showed that urine could be an noninvasive way to diagnose toxoplasmosis by detecting antigens or identifying *T*. *gondii* DNA. Other tests like RLA and coagglutination may ease finding the antigen in urine, whereas nested PCR might be effective for detecting DNA. Still, these results should not be seen as a reason to replace diagnostic methods. However, the reviewed studies had limitations. Including different sample sizes and there were differences in how participants were selected, which tests were used and how the testing was carried out. These differences make it hard to compare results across studies. Limit how broadly the findings can be applied. Because of this, it is early to say which test works best. There are also concerns. Different parasite strains may affect test results. PCR might miss the infection sometimes leading to negative results, and there is also a risk of positives due to contamination. That is why it is important to handle samples in a way optimize the assays and use proper quality controls. Another issue is the lack of long‐term studies and enough clinical testing to back up real‐world usefulness. More scale, well‐designed studies are needed. These should use the procedures and reference standards to properly assess how well urine‐based tests work in different settings. Then can we know if urine tests can reasonably support current diagnostic pathways.

## Author Contributions

Conceptualization: A.D. and R.A.; data curation: S.A.H., R.A., and T.N.; formal analysis: S.A.H.; investigation: R.A. and T.N.; methodology: A.D., T.N., and S.A.; project administration: A.D. and S.A.H.; software: S.A.H.; supervision: A.D., S.A.H., and S.S.; validation: A.D., T.N., S.A., S.A.H., S.S., and S.G.; writing—original draft: R.A., T.N., and S.A.H.; writing—review and editing: S.A.H., T.N., B.B., and M.S.

## Funding

No funding was received for this manuscript.

## Ethics Statement

No animals/humans were used for studies that are the basis of this research.

## Consent

The authors have nothing to report.

## Conflicts of Interest

The authors declare no conflicts of interest.

## Data Availability

The data that support the findings of this study are available from the corresponding authors upon reasonable request.
